# Modeling branching in cereals

**DOI:** 10.3389/fpls.2013.00399

**Published:** 2013-10-10

**Authors:** Jochem B. Evers, Jan Vos

**Affiliations:** Centre for Crop Systems Analysis, Wageningen UniversityWageningen, Netherlands

**Keywords:** functional–structural plant modeling, cereal, grass, branching, tillering, tillering probability, dose–response curve, mechanistic modeling

## Abstract

Cereals and grasses adapt their structural development to environmental conditions and the resources available. The primary adaptive response is a variable degree of branching, called tillering in cereals. Especially for heterogeneous plant configurations the degree of tillering varies per plant. Functional–structural plant modeling (FSPM) is a modeling approach allowing simulation of the architectural development of individual plants, culminating in the emergent behavior at the canopy level. This paper introduces the principles of modeling tillering in FSPM, using (I) a probability approach, forcing the dynamics of tillering to correspond to measured probabilities. Such models are particularly suitable to evaluate the effect structural variables on system performance. (II) Dose–response curves, representing a measured or assumed response of tillering to an environmental cue. (III) Mechanistic approaches to tillering including control by carbohydrates, hormones, and nutrients. Tiller senescence is equally important for the structural development of cereals as tiller appearance. Little study has been made of tiller senescence, though similar concepts seem to apply as for tiller appearance.

## INTRODUCTION

Production of branches (tillering) is an important trait of many cereal plants such as wheat (*Triticum aestivum*) and rice (*Oryza* species). Cereal plants are able to maximize total plant light capture and grain production through processes such as bud dormancy break, tiller development, and tiller senescence. These processes are highly plastic: the growing conditions a cereal plant experiences strongly influence the tillering characteristics of the plant (e.g., Casal et al., 1990; Rodríguez et al., 1999; Lafarge and Hammer, 2002; Evers et al., 2006; Sparkes et al., 2006). At high population densities, bud break is generally low and tiller mortality is relatively high (Darwinkel, 1978).

Most crop growth models of cereals, which aim at predicting grain production on an area basis, do not take into consideration the plant’s response to environmental conditions in terms of tiller production (Jamieson et al., 1998). For many scenarios this is not a problem, since within a common range of agronomical practice (population density, row distance) leaf area and ear production is rather predictable and stable when expressed per unit of ground area. However, accurate prediction of variables such as light interception and ear production becomes more difficult in the case of more heterogeneous canopy configurations, such as in intercropping systems (Li et al., 2001), wide-row crop systems (Winter and Welch, 1987), and in crops that show erratic emergence and establishment. Such non-uniform leaf area distribution is difficult to represent in most crop models, leading to inaccuracies in predictions of crop growth.

Here, we review the possibilities to simulate branch production in cereals using a plant architectural modeling technique: functional–structural plant modeling (FSPM; Vos et al., 2010; DeJong et al., 2011; Evers et al., 2011). Using FSPM, tiller production and senescence can be evaluated for every individual plant in the canopy. This results in an accurate three-dimensional representation of canopy development over time. In this paper, we show how tiller appearance and senescence can be represented in FSPM and how internal and environmental regulation of tillering can be implemented.

## MODELING CEREAL ARCHITECTURE

Leaves are provided with tiller buds in their axils, which only produce a branch if circumstances are favorable. Therefore, the composition of the vegetative cereal phytomer is always the same: an internode, a leaf (sheath and lamina), and an axillary bud (McMaster, 2005; Forster et al., 2007). Modeling cereal architecture starts with the phytomer which, in classic L-system notation (Prusinkiewicz and Lindenmayer, 1990), can be represented by a string of characters B (tiller bud), I (internode), N (node), S (sheath) and L (lamina):

[B]IN[SL]

where the brackets represent structures forking off the main axis such as leaves and branches. A typical L-system rewriting rule (Prusinkiewicz and Lindenmayer, 1990) that represents the creation of new phytomers by the apical meristem (A) is:

(1) A⇒[B]IN[SL]A

**FIGURE 1:**
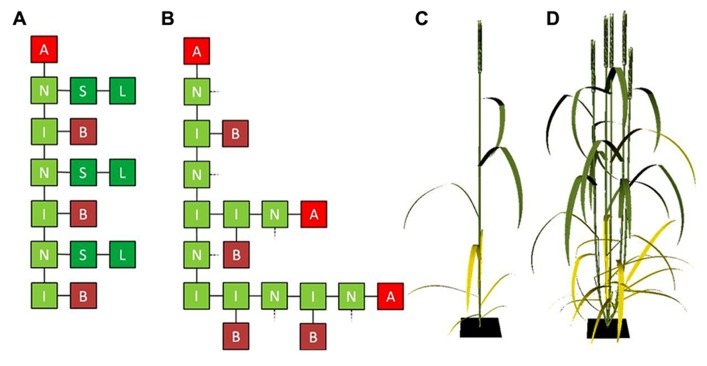
**(A)** Graphical representation of an L-system string containing three vegetative cereal phytomers with an apical meristem on top and **(B)** three vegetative cereal phytomers the lower two of which have grown a two-phytomer and one-phytomer tiller, respectively. L, lamina; S, sheath; I, internode; N, node; B, bud; A, apical meristem. In **(B)**, sheaths and laminae (connected at the dashed lines) have been omitted for clarity. **(C,D)** Cereal architecture simulated using L-systems, using functional–structural plant modeling (FSPM): cereal plant in flowering stage with no tillers **(C)** and with four tillers **(D)**.

Starting with only A, and applying the rewriting rule three times will result in a stem segment consisting of three phytomers and a shoot apical meristem at the top, represented by the string:

[B]IN[SL][B]IN[SL][B]IN[SL]A

which could be represented graphically as shown in **Figure 1A**. As the shoot develops and under favorable conditions, cereal shoots produce tillers in acropetal direction. An L-system rewriting rule that represents the change from a dormant bud to an actively developing shoot could simply look like:

(2) B⇒A

after which rule 1 could be applied to the newly created apex, to make the tiller develop like its parent shoot. In most cases tillering starts from the bottom-most phytomer, which is represented in **Figure 1B** for the case of a developing four-phytomer shoot with two developing tillers. In turn, the buds present on the first-order tillers can potentially produce tillers themselves. In this way higher-order tillers, which frequently occur in cereals, can be generated.

The representation of tillering above only considers the network of interconnected organs, i.e., the topology of the plant. To be able to simulate regulation of tiller appearance and senescence by internal and/or environmental factors, organ geometry needs to be considered as well. Geometrical characteristics such as internode length, blade size, shape and angle, shoot and leaf orientation determine factors like transport of compounds throughout the plant, and interception and scattering of light by the plant’s organs. In FSPM, organ geometry can be taken into account explicitly, which, together with plant topology, allows for accurate three-dimensional representation of plant architecture (**Figures 1C,D**).

## MODELING REGULATION OF TILLERING

### PROBABILITY DISTRIBUTIONS

The number of tillers formed and senesced can be represented in an FSP model using a purely statistical, descriptive approach. To this end, each bud represented in the model is typically provided with a value for the probability it will break and form a tiller, and the probability it will senesce before reaching maturity. At initiation of each bud the values for these parameters are chosen randomly from a distribution of values obtained experimentally. Typically, such distributions are determined for a range of population densities, nutrition levels or light levels. As such conditions are normally model input, an appropriate number of tillers will emerge upon model execution, mimicking tillering in real canopies (Watanabe et al., 2005; Evers et al., 2007b). This is fine in those cases where plant stands experiencing one certain set of conditions is being simulated, for example, for a particular population density or climate. Such simulated copies of real plant stands can subsequently be used, e.g., to assess the impact of cultivar leaf angle on rate soil covering, the light climate within the canopy during cereal crop development, the dispersion of fungi within a crop canopy, etc.

However, modeling of tillering using probability distributions becomes more cumbersome and less useful in case canopy configuration or environmental conditions are not uniform on an area basis. Such models based on single parameter distributions cannot represent tillering characteristics of border plants, especially in intercropping and wide-row systems. A solution could be to determine the local conditions per plant and provide the model with parameter distributions for all sets of local conditions occurring. A more elegant and simple solution to this problem is to use dose–response curves directly relating environment to tillering.

### DOSE–RESPONSE CURVES

**FIGURE 2:**
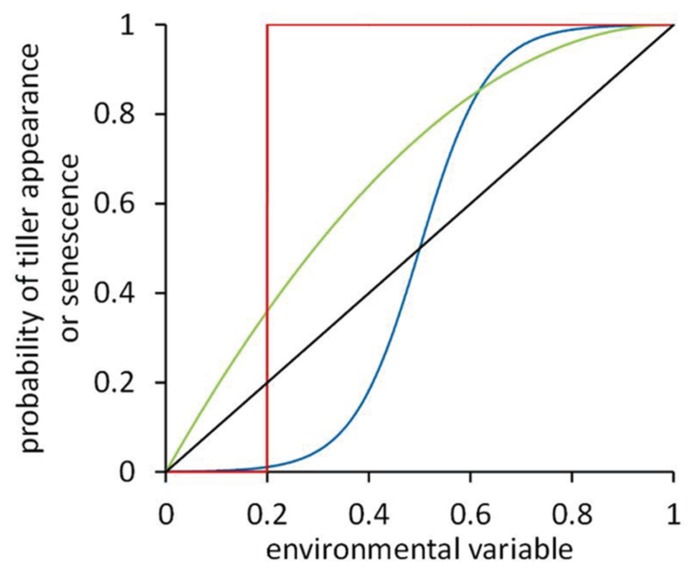
**Dose–response curves representing the response of tiller appearance or senescence probability to some environmental variable.** Four hypothetical curves are shown: unit-step response with a threshold value of 0.2 (red line), a curvilinear response (green line), a sigmoidal response with an inflection point at 0.5 (blue line), and a linear response (black line).

Tiller bud break and tiller senescence are known to directly depend on environmental conditions, such as soil phosphorus (e.g., Rodríguez et al., 1999; Dingkuhn et al., 2006) and nitrogen (Zhong et al., 2003; Alzueta et al., 2012), and the red/far-red ratio (R:FR) of the light within the canopy (e.g., Casal et al., 1987; Sparkes et al., 2006). To accurately describe the tillering response of cereal plants to local light or nutrient conditions in an FSP model of cereal development, dose–response curves can be implemented. In FSPM such curves relate an environmental variable such as R:FR (Evers et al., 2007a) or multiple environmental variables such as both R:FR and light intensity (Gautier et al., 2000) to the probability of a tiller to start growing or to senesce. The shape of such a curve depends on the response observed experimentally. Dose–response curves may have diverse shapes (**Figure 2**).

In the case of light, an essential difference between models using probability distributions and those using response curves relating light to tillering is that the latter allow for tiller-environment feedback. Newly formed tillers and tillers that just senesced affect the light environment, possibly affecting appearance and senescence of other tillers on the same or neighboring plants. This feedback between tillering and the light climate in a canopy gives interesting opportunities for research questions in the domain of plant manipulation or other processes affecting plant architecture. Processes such as defoliation, thinning, or (partial) plant death due to diseases can be implemented in the FSP model, and the resulting effects on tillering behavior can be studied.

Dose–response curves enable the simulated plants to make their tillering behavior depend on local conditions. Plants at the border of a simulated plot will experience a different nutritional status of the soil (less belowground competition) and/or a different light climate (higher radiation intensity, higher R:FR), and will consequently produce more tillers compared to plants in the middle of the plot. Depending on which type of response curve was chosen, simulated tillering behavior may or may not realistically mimic actual observations (Evers et al., 2007a). Nevertheless, models simulating tiller appearance and senescence using response curves still merely *describe* tillering behavior rather than *explain* it. For research questions that focus on understanding how tillering is regulated, and what processes are involved and are interacting to result in the tillering patterns observed, another level of detail needs to be added.

### MECHANISTIC MODELING OF TILLER APPEARANCE

The term mechanistic modeling is used for those models that incorporate mechanisms on one level of integration, and provide output at a higher level of integration. Such models aim at explaining the output based on the underlying mechanisms. Therefore, mechanistic models are usually capable of predicting also outside the ranges they were originally calibrated for. Tillering is controlled through many different mechanisms (Tomlinson and O’Connor, 2004; McSteen, 2009; Assuero and Tognetti, 2010). Here, we will consider three main groups of processes related to tillering control (regulation by carbohydrate availability, by hormones, and by macronutrients) to discuss mechanistic modeling of tillering.

#### Carbohydrate control

A bud needs carbohydrates to grow out into a tiller, making it a strong sink for carbohydrates. In case a plant experiences low light levels, or has many sink organs simultaneously, the ratio between the supply and demand for carbohydrates (the source/sink ratio) may be low. In such a case only a fraction of the buds will have the opportunity to grow a tiller. As a new tiller develops, it gradually changes its role from sink to source for carbohydrates, influencing the source/sink ratio of the whole plant. Next to their role as substrates for growth, carbohydrates have also been identified as signaling molecules for a host of physiological processes (sugar signaling; Rolland et al., 2006) which may affect tillering. Although most evidence of tillering control by carbohydrates either as growth substrates or as physiological signals is of correlative nature (Assuero and Tognetti, 2010), carbohydrates are undeniably needed for branch growth, so the source/sink ratio has been implemented widely in simulation models as a determinant of tillering and branching (Luquet et al., 2006; Tomlinson et al., 2007; Mathieu et al., 2009; Evers et al., 2010).

To implement carbohydrate control of tillering in an FSP model, processes related to carbohydrate supply and demand need to be incorporated. Carbohydrates supply is usually captured by implementing light absorption and photosynthesis routines at the level of the plant organ (Wernecke et al., 2007; Evers et al., 2010; Xu et al., 2011). Light absorption can be calculated using various approaches such as radiosity or ray-tracing (Chelle and Andrieu, 1999), which take into account reflection, transmission, and absorption of photosynthetically active radiation by all organs in the simulated canopy. The most popular photosynthesis sub-model in FSPM and many other types of plant and crop model is the Farquhar–von Caemmerer–Berry (FvCB) biochemical photosynthesis model (Farquhar et al., 1980). The FvCB model can be calibrated easily using data from gas-exchange measurements. In FSPM, light absorption and photosynthesis simulation give carbohydrate supply at the organ level, which may differ between organs depending on their local light environment. A frequently used approach to modeling carbohydrate demand at the organ scale is the relative sink-strength approach (Heuvelink, 1996) which dictates that substrates are allocated to growing organs according to their relative sink strength, i.e., their potential growth rate (in units of substrate demanded per unit of time) proportional to the potential growth rate of the whole plant. The ratio between the total plant supply of carbohydrates as calculated from organ photosynthesis, and the total plant demand for carbohydrates calculated as the sum of the potential growth rates of all organs requiring carbohydrates, is the source/sink ratio.

Instead of attempting to estimate the sink strength of individual buds, a threshold value of the source/sink ratio is often determined above which buds are allowed to form a tiller. Such a threshold may represent a physiological state analogous to sugar signaling (Luquet et al., 2006). A threshold value of 1.0 means that a tiller may develop in case the carbohydrate supply exceeds the demand. Lower or higher values may represent more opportunistic or conservative strategies toward tiller development, respectively.

#### Hormonal control

A complex system of hormonal interactions controls branch formation in general (Leyser, 2009; Domagalska and Leyser, 2011; Dun et al., 2012). To a large extent tillering is governed by the same processes, although there are small differences compared to dicots (McSteen, 2009; Assuero and Tognetti, 2010). Processes in branching control concern (long-distance) signaling by plant hormones auxin, strigolactone (both branching suppressors), and cytokinin (branching promotor) and are conserved between mono- and dicots. In grasses, ethylene and gibberellins also play a role (Rajala and Peltonen-Sainio, 2001; Frantz et al., 2004; Kebrom et al., 2013).

In order to simulate hormonal control of tillering using FSPM, processes such as hormone biosynthesis, transport, and decay need to be implemented. In their pioneering work, Prusinkiewicz et al. (2009) associated biosynthesis of auxin with modules representing the apex and the buds in a simple FSP model, and incorporated routines to calculate active (i.e., transporter-protein mediated) transport of auxin through the developing plant structure. Bud activation and subsequent branch development was an emerging property of the model, driven by auxin levels in the bud and the adjacent stem and by the feedback between the dynamics of auxin and transport-protein levels. This approach was adopted and extended to simulate R:FR control of hormone-regulated branching in *Arabidopsis* (Evers and Van der Krol, 2012). Similar approaches could be used to simulate cereal tillering as well. The current discussion on which hormonal factors are involved for branching control in grasses and dicots (Dun et al., 2012; Renton et al., 2012; Shinohara et al., 2013) provide good opportunities for FSPM to test hypotheses on branching and tillering control.

#### Nutrient control

Both soil nitrogen and phosphorus affect tillering in cereals (Rodríguez et al., 1999; Zhong et al., 2003; Dingkuhn et al., 2006; Alzueta et al., 2012). Soil nitrogen limitation can suppress branch growth directly (McIntyre, 2001) and through an effect on production of cytokinin (Tomlinson and O’Connor, 2004). Soil phosphorus limitation results in decreased branching (Kohlen et al., 2011) acting through hormone signaling by stimulating strigolactone production and transport within the plant.

Analogous to control by carbohydrates, simulation of tillering control by nutrients requires definition of nutrient supply, nutrient demand, and allocation of nutrients to demanding organs. Simulation of nutrient supply, i.e., uptake by the root system, itself can be done at various levels of detail. The simplest approach is to provide the simulated plant with nutrients each time step according to measured values of uptake. A far more elaborate approach is to include the soil environment and development and growth of the root system architecture, making nutrient uptake dependent on root architecture, rooting depth and horizontal distribution, heterogeneity in soil nutrient distribution, uptake processes, etc. (Dunbabin et al., 2004; Pagès et al., 2004). In principle, nutrient demand and allocation can be included similar to carbohydrates, which would allow for simulation of both tiller production and tiller senescence.

## MECHANISTIC MODELING OF TILLER SENESCENCE

Upon cessation of appearance of new tillers a phase sets in of cessation of growth and onset of senescence of part of the tillers. The number of appeared tillers represents an adaptation to the environment. A variable fraction of survival is another adaptation option, occurring somewhat later in the life cycle than cessation of tiller appearance. As mentioned, the same modeling paradigms can be applied to tiller senescence as to cessation of tiller appearance, i.e., from probability distributions, dose–response curves up to mechanistic modeling. Sparkes et al. (2006) associated the onset of tiller senescence with the drop below a critical value of R:FR ratio at the base of the canopy. Interestingly, this critical R:FR threshold was suggested to interact with leaf nitrogen content – where leaf nitrogen content is higher, the critical R:FR is lower. In other words, when more nitrogen is available, the canopy is allowed to grow larger before tiller death starts and vice versa. The carbohydrate source/sink ratio may proof to be a suitable concept to simulate tiller senescence but to our knowledge this has not been studied. Similar remarks apply to hormonal and nutrient control. For good reasons research has addressed mechanisms governing branching and tillering but for realistic modeling of the architectural dynamics of plants it is equally important to develop our understanding of the processes that govern senescence of tillers and branches.

## CONCLUDING REMARKS

FSP models provide excellent opportunities to address questions related to tillering in cereals, its regulation, environmental response, and consequences at plant and canopy level. Explicitly including tillering in a model may improve predictions of leaf area development especially in non-uniform canopies such as those in intercropping or wide-row systems. The choice whether to simulate tillering using probabilities, driven by dose–response relationships or by underlying processes depends very much on the purpose of the modeling exercise. If the goal is to mimic the three-dimensional structure of a cereal canopy, to be used for instance in a light-interception study, modeling of tiller appearance and senescence using probabilities may be sufficient. When studying the dynamics of tillering itself, it is essential to include the feedback between environment and tillering in the model. In such cases dose–response curves or more mechanistic approaches are required, which have disadvantages of additional data requirement and computational costs. In any case, FSP models are capable of simulating tillering and the consequences for cereal architecture at a high level of detail using well-established and straightforward modeling techniques. As such, FSP models can seamlessly complement experimental studies on plant and canopy development.

## Conflict of Interest Statement

The authors declare that the research was conducted in the absence of any commercial or financial relationships that could be construed as a potential conflict of interest.

## References

[B1] Alzueta I. Abeledo L. G. Mignone C. M. Miralles D. J. (2012). Differences between wheat and barley in leaf and tillering coordination under contrasting nitrogen and sulfur conditions. *Eur. J. Agron.* 41 92–102 10.1016/j.eja.2012.04.002

[B2] Assuero S. G. Tognetti J. A. (2010). Tillering regulation by endogenous and environmental factors, and its agricultural management. *Am. J. Plant Sci. Biotechnol.* 4 35–48

[B3] Casal J. J. Sánchez R. A. Deregibus V. A. (1987). Tillering responses of *Lolium multiflorum* plants to changes of red/far-red ratio typical of sparse canopies. *J. Exp. Bot.* 38 1432–1439 10.1093/jxb/38.9.1432

[B4] Casal J. J. Sánchez R. A. Gibson D. (1990). The significance of changes in the red/far-red ratio, associated with either neighbour plants or twilight, for tillering in *Lolium multiflorum* Lam. *New Phytol.* 116 565–572 10.1111/j.1469-8137.1990.tb00540.x

[B5] Chelle M. Andrieu B. (1999). Radiative models for architectural modelling. *Agronomie* 19 225–240 10.1051/agro:19990304

[B6] Darwinkel A. (1978). Patterns of tillering and grain production of winter wheat at a wide range of plant densities. *Neth. J. Agric. Sci.* 26 383–398

[B7] DeJong T. M. Da Silva D. Vos J Escobar-Gutiérrez A. J. (2011). Using functional–structural plant models to study, understand and integrate plant development and ecophysiology. *Ann. Bot.* 108 987–989 10.1093/aob/mcr257 22084818 PMC3189848

[B8] Dingkuhn M. Luquet D. Kim H. Tambour L. Clement-Vidal A. (2006). EcoMeristem, a model of morphogenesis and competition among sinks in rice. 2. Simulating genotype responses to phosphorus deficiency. *Funct. Plant Biol.* 33 325–337 10.1071/FP05267 32689239

[B9] Domagalska M. A. Leyser O. (2011). Signal integration in the control of shoot branching. *Nat. Rev. Mol. Cell Biol.* 12 211–221 10.1038/nrm3088 21427763

[B10] Dun E. A. De Saint Germain A. Rameau C. Beveridge C. A. (2012). Antagonistic action of strigolactone and cytokinin in bud outgrowth control. *Plant Physiol.* 158 487–498 10.1104/pp.111.186783 22042819 PMC3252097

[B11] Dunbabin V. Rengel Z. Diggle A. J. (2004). Simulating form and function of root systems: efficiency of nitrate uptake is dependent on root system architecture and the spatial and temporal variability of nitrate supply. *Funct. Ecol.* 18 204–211 10.1111/j.0269-8463.2004.00827.x

[B12] Evers J. B Van der Krol A. R. (2012). “Capturing hormonal and light interactions in a simulation model of shoot branching,” in *Plant Growth Modelling, Simulation, Visualization and Applications - PMA12* eds Kang M. Dumont Y. Guo Y. ([Beijing: IEEE) 101–108

[B13] Evers J. B. Van Der Krol A. R. Vos J. Struik P. C. (2011). Understanding shoot branching by modelling form and function. *Trends Plant Sci.* 16 464–467 10.1016/j.tplants.2011.05.004 21658989

[B14] Evers J. B. Vos J. Andrieu B. Struik P. C. (2006). Cessation of tillering in spring wheat in relation to light interception and red:far-red ratio. *Ann. Bot.* 97 649–658 10.1093/aob/mcl020 16464875 PMC2803660

[B15] Evers J. B. Vos J. Chelle M. Andrieu B. Fournier C. Struik P. C. (2007a). Simulating the effects of localized red:far-red ratio on tillering in spring wheat (*Triticum aestivum*) using a three-dimensional virtual plant model. *New Phytol.* 176 325–336 10.1111/j.1469-8137.2007.02168.x 17888114

[B16] Evers J. B. Vos J. Fournier C. Andrieu B. Chelle M. Struik P. C. (2007b). An architectural model of spring wheat: evaluation of the effects of population density and shading on model parameterization and performance. *Ecol. Model.* 200 308–320 10.1016/j.ecolmodel.2006.07.042

[B17] Evers J. B. Vos J. Yin X. Romero P. Van Der Putten P. E. L. Struik P. C. (2010). Simulation of wheat growth and development based on organ-level photosynthesis and assimilate allocation. *J. Exp. Bot.* 61 2203–2216 10.1093/jxb/erq025 20231326

[B18] Farquhar G. D. von Caemmerer S. Berry J. A. (1980). A biochemical model of photosynthetic CO_2_ assimilation in leaves of C_3_ species. *Planta* 149 78–90 10.1007/BF00386231 24306196

[B19] Forster B. P. Franckowiak J. D. Lundqvist U. Lyon J. Pitkethly I Thomas W. T. B. (2007). The barley phytomer. *Ann. Bot.* 100 725–733 10.1093/aob/mcm183 17901062 PMC2749621

[B20] Frantz J. M. Pinnock D. Klassen S. Bugbee B. (2004). Characterizing the environmental response of a gibberellic acid-deficient rice for use as a model crop. *Agron. J.* 96 1172–1181 10.2134/agronj2004.1172

[B21] Gautier H. Mĕch R. Prusinkiewicz P. Varlet-Grancher C. (2000). 3D architectural modelling of aerial photomorphogenesis in white clover (*Trifolium repens* L.) using L-systems. * Ann. Bot. * 85 359–370 10.1006/anbo.1999.1069

[B22] Heuvelink E. (1996). Dry matter partitioning in tomato: validation of a dynamic simulation model. *Ann. Bot.* 77 71–80 10.1006/anbo.1996.0009

[B23] Jamieson P. D. Porter J. R. Goudriaan J. Ritchie J. T. Van Keulen H. Stol W. (1998). A comparison of the models AFRCWHEAT2, CERES-Wheat, Sirius, SUCROS2 and SWHEAT with measurements from wheat grown under drought. *Field Crops Res.* 55 23–44 10.1016/S0378-4290(97)00060-9

[B24] Kebrom T. H. Spielmeyer W. Finnegan E. J. (2013). Grasses provide new insights into regulation of shoot branching. *Trends Plant Sci.* 18 41–48 10.1016/j.tplants.2012.07.001 22858267

[B25] Kohlen W. Charnikhova T. Liu Q. Bours R. Domagalska M. A. Beguerie S. (2011). Strigolactones are transported through the xylem and play a key role in shoot architectural response to phosphate deficiency in non-AM host *Arabidopsis thaliana*. *Plant Physiol.* 155 974–987 10.1104/pp.110.164640 21119045 PMC3032481

[B26] Lafarge T. A. Hammer G. L. (2002). Tillering in grain sorghum over a wide range of population densities. Modelling dynamics of tiller fertility. *Ann. Bot.* 90 99–110 10.1093/aob/mcf153 PMC4233857 12125777

[B27] Leyser O. (2009). The control of shoot branching: an example of plant information processing. *Plant Cell Environ.* 32 694–703 10.1111/j.1365-3040.2009.01930.x 19143993

[B28] Li L. Sun J. Zhang F. Li X. Yang S. Rengel Z. (2001). Wheat/maize or wheat/soybean strip intercropping: I. Yield advantage and interspecific interactions on nutrients.* Field Crops Res.* 71 123–137 10.1016/S0378-4290(01)00156-3

[B29] Luquet D. Dingkuhn M. Kim H. Tambour L. Clement-Vidal A. (2006). EcoMeristem, a model of morphogenesis and competition among sinks in rice. 1. Concept, validation and sensitivity analysis. *Funct. Plant Biol.* 33 309–323 10.1071/FP05266 32689238

[B30] Mathieu A. Cournede P. H. Letort V. Barthelemy D De Reffye P. (2009). A dynamic model of plant growth with interactions between development and functional mechanisms to study plant structural plasticity related to trophic competition. *Ann. Bot.* 103 1173–1186 10.1093/aob/mcp054 19297366 PMC2685317

[B31] McIntyre G. I. (2001). Control of plant development by limiting factors: a nutritional perspective. *Physiol. Plant.* 113 165–175 10.1034/j.1399-3054.2001.1130203.x 12060293

[B32] McMaster G. S. (2005). Phytomers, phyllochrons, phenology and temperate cereal development. *J. Agric. Sci.* 143 137–150 10.1017/S0021859605005083

[B33] McSteen P. (2009). Hormonal regulation of branching in grasses. *Plant Physiol.* 149 46–55 10.1104/pp.108.129056 19126694 PMC2613715

[B34] Pagès L. Vercambre G. Drouet J.-L. Lecompte F. Collet C Le Bot J. (2004). Root Typ: a generic model to depict and analyse the root system architecture. *Plant Soil* 258 103–119 10.1023/B:PLSO.0000016540.47134.03

[B35] Prusinkiewicz P. Crawford S. Smith R. S. Ljung K. Bennett T. Ongaro V. (2009). Control of bud activation by an auxin transport switch. *Proc. Natl. Acad. Sci. U.S.A.* 106 17431–17436 10.1073/pnas.0906696106 19805140 PMC2751654

[B36] Prusinkiewicz P. Lindenmayer A. (1990). *The Algorithmic Beauty of Plants*. New York: Springer-Verlag 10.1007/978-1-4613-8476-2

[B37] Rajala A. Peltonen-Sainio P. (2001). Plant growth regulator effects on spring cereal root and shoot growth. *Agron. J.* 93 936–943 10.2134/agronj2001.934936x

[B38] Renton M. Hanan J. Ferguson B. J. Beveridge C. A. (2012). Models of long-distance transport: how is carrier-dependent auxin transport regulated in the stem? *New Phytol.* 194 704–715 10.1111/j.1469-8137.2012.04093.x 22443265

[B39] Rodrïguez D. Andrade F. H. Goudriaan J. (1999). Effects of phosphorus nutrition on tiller emergence in wheat. *Plant Soil* 209 283–295 10.1023/A:1004690404870

[B40] Rolland F. Baena-Gonzalez E. Sheen J. (2006). Sugar sensing and signaling in plants: conserved and novel mechanisms. *Annu. Rev. Plant Biol.* 57 675–709 10.1146/annurev.arplant.57.032905.105441 16669778

[B41] Shinohara N. Taylor C. Leyser O. (2013). Strigolactone can promote or inhibit shoot branching by triggering rapid depletion of the auxin efflux protein PIN1 from the plasma membrane. *PLoS Biol. * 11:e1001474 10.1371/journal.pbio.1001474 PMC3558495 23382651

[B42] Sparkes D. L. Holme S. J. Gaju O. (2006). Does light quality initiate tiller death in wheat? *Eur. J. Agron.* 24 212–217 10.1016/j.eja.2005.08.003

[B43] Tomlinson K. W. Dominy J. G. Hearne J. W O’Connor T. G. (2007). A functional–structural model for growth of clonal bunchgrasses. *Ecol. Model.* 202 243–264 10.1016/j.ecolmodel.2006.11.002

[B44] Tomlinson K. W O’Connor T. G. (2004). Control of tiller recruitment in bunchgrasses: uniting physiology and ecology. *Funct. Ecol.* 18 489–496 10.1111/j.0269-8463.2004.00873.x

[B45] Vos J. Evers J. B. Buck-Sorlin G. H. Andrieu B. Chelle M De Visser P. H. B. (2010). Functional–structural plant modelling: a new versatile tool in crop science. *J. Exp. Bot.* 61 2102–2115 10.1093/jxb/erp345 19995824

[B46] Watanabe T. Hanan J. S. Room P. M. Hasegawa T. Nakagawa H. Takahashi W. (2005). Rice morphogenesis and plant architecture: measurement, specification and the reconstruction of structural development by 3D architectural modelling. *Ann. Bot.* 95 1131–1143 10.1093/aob/mci136 15820987 PMC4246908

[B47] Wernecke P. Müller J. Dornbusch T. Wernecke A. Diepenbrock W. (2007). “The virtual crop-modelling system “VICA” specified for barley,” in *Functional–Structural Plant Modelling in Crop Production* eds Vos J. Marcelis L. F. M. De Visser P. H. B. Struik P. C. Evers J. B. (Dordrecht: Springer) 53–64

[B48] Winter S. R. Welch A. D. (1987). Tall and semidwarf wheat response to dryland planting systems. *Agron. J.* 79 641–645 10.2134/agronj1987.00021962007900040012x

[B49] Xu L. Henke M. Zhu J. Kurth W. Buck-Sorlin G. (2011). A functional–structural model of rice linking quantitative genetic information with morphological development and physiological processes. *Ann. Bot.* 107 817–828 10.1093/aob/mcq264 21247905 PMC3077984

[B50] Zhong X. Peng S. Sanico A. L. Liu H. (2003). Quantifying the interactive effect of leaf nitrogen and leaf area on tillering of rice. *J. Plant Nutr.* 26 1203–1222 10.1081/PLN-120020365

